# Effects of water extracts of *Flaveria bidentis* on the seed germination and seedling growth of three plants

**DOI:** 10.1038/s41598-022-22527-z

**Published:** 2022-10-21

**Authors:** Lei Dai, Lili Wu, Xiuren Zhou, Zaiyou Jian, Li Meng, Guifang Xu

**Affiliations:** 1 Henan Institute of Science and Technology, Postdoctoral Research Base, Xinxiang, 453003 China; 2 College of Life Sciences, Henan Normal University, Xinxiang, 453007 China

**Keywords:** Ecology, Physiology, Plant sciences

## Abstract

To further explore the mechanism behind the allelopathic effects of *Flaveria bidentis*, we investigated the allelopathic effects of water extracts from *Flaveria bidentis* leaves on three plants, Shanghai green, barnyard grass and wheat. The results showed that the water extracts inhibited the germination potential, germination rate, seedling height, root length, chlorophyll content, fresh weight and dry weight of the three plants, and increasing the extract concentration further increased the inhibitory effect. The allelopathic effects of the water extracts from *Flaveria bidentis* leaves on the three receptor plants differed in strength from strong to weak as follows: Shanghai green > barnyard grass > wheat. Thus, wheat had strong resistance to the allelopathic effects of *Flaveria bidentis* and could be planted in area where *Flaveria bidentis* occurs. The effect of the water extract from *Flaveria bidentis* leaves on the seed germination and seedling growth of barnyard grass was obvious; thus, this extract could be used for the biological control of barnyard grass.

## Introduction

*Flaveria bidentis*, also called bidentate yellow chrysanthemum, belongs to the Asteraceae genus Flaveria, originating from South America. This plant was first discovered near Nankai University in Tianjin, China, in 2001^1–3^. *Flaveria bidentis* grows mostly in wasteland areas and has light-loving, salt-tolerant, fast-growing, large-bodied, strongly adaptable, and rapidly spreading characteristics. This plant is included on the list of quarantine pests from imported plants in China^4–6^.

Plant allelopathy means that plants release allelopathic substances into their surroundings through leaching, root secretion and the degradation of residues; these substances may have direct or indirect beneficial or harmful effects on surrounding plants^7–9^. Allelopathy is considered to be one of the potential mechanisms for the successful invasion of exotic plants into new habitats^10–14^. Bais et al.^15^ and Hu et al.^16^ believed that allelopathy plays a very important role in biological invasion.

There have been many studies on the allelopathic effects of *Flaveria bidentis*. For example, Lu et al. studied the allelopathic effects of *Flaveria bidentis* on two kinds of vegetables^17^. The results showed that *Flaveria bidentis* extracts had a significant inhibitory effect on the germination of *Lactuca sativa* and could also significantly depress the root length and seedling height of *Brassica napus* and *Lactuca sativa*. Studies on the allelopathic effect of *Flaveria bidentis* on herbage showed that the yellow-top root residues were more inhibitory than leaf residues, thus explaining the residual effects of these substances on the surrounding soil^18^. Another study found that the residues of *Flaveria bidentis* had an inhibitory effect on the early growth of cotton, and *Flaveria bidentis* released allelochemicals to the soil to affect the fertility of the soil^19^.

Ji et al. showed that the germination rate of cucumber seeds decreased as the concentration of the *Flaveria bidentis* extract increased^20^. Some active substances in the root exudates of *Flaveria bidentis* had inhibitory effects on the seed germination, seedling growth and root length of *Triticum aestivum*, *Cucumis sativus*, and *Amaranthus retroflexus*^21^. Yan et al*.* indicated that the water extracts of *Flaveria bidentis* had different inhibitory effects on the growth of four crops (corn, wheat, soybean and cabbage)^22^. Although there have been some studies on the allelopathic effects of *Flaveria bidentis*, there have been no reports about the allelopathy of *Flaveria bidentis* on Shanghai green, wheat and barnyard grass. Thus, we selected Shanghai green, barnyard grass and wheat as the receptors to study the allelopathic effect of different concentrations of *Flaveria bidentis* leaf extracts on the seeds of these three plants. Our goal was to explore the allelopathic mechanism of *Flaveria bidentis,* to identify crop species resistant to allelopathy, and to provide a scientific reference for the allelopathic control of weeds.

## Results

### Effect of *Flaveria bidentis* leaf extracts on the germination of three receptor seeds

The water extract from the leaves of *Flaveria bidentis* had significant inhibitory effects (RI < 0) on the germination potential and germination rate of three plants, Shanghai green, wheat and barnyard grass, and the inhibition increased as the concentration increased (Table 1). At a concentration of 0.025 g/mL, the germination potential and germination rate of the three plants were significantly different from those of the control. When the concentration was 0.100 g/mL, the germination inhibition of the three plants was the strongest. At this time, the germination potential and germination rate of Shanghai green and barnyard grass decreased to 0, and the germination potential and germination rate of wheat decreased by approximately 87% and 75% compared with the control, respectively (Table 1).

**Table 1:** Effects of the water extract from leaves of *Flaveria bidentis* on the seed germination potential and germination rate of three plants.

Plant species	Concentration (g/mL)	Germination potential (%)	RI of the germination potential	Germination rate (%)	RI of the germination rate
Shanghai green	0	72.00 ± 5.12a		80.67 ± 3.13a	
	0.025	28.67 ± 2.73b	− 0.60	54.00 ± 2.12b	− 0.33
	0.050	9.33 ± 0.14c	− 0.87	11.33 ± 1.34c	− 0.86
	0.075	3.33 ± 0.02d	− 0.95	5.33 ± 0.23d	− 0.93
	0.100	0.00 ± 0.00e	− 1.00	0.00 ± 0.00e	− 1.00
Wheat	0	75.00 ± 3.05a		86.67 ± 5.32a	
	0.025	48.33 ± 2.23b	− 0.36	60.00 ± 3.17b	− 0.31
	0.050	31.67 ± 1.56c	− 0.58	41.67 ± 2.21c	− 0.52
	0.075	20.00 ± 1.25d	− 0.73	28.33 ± 1.58d	− 0.67
	0.100	10.00 ± 0.16e	− 0.87	21.67 ± 1.14e	− 0.75
Barnyard grass	0	70.00 ± 3.56a		81.11 ± 4.16a	
	0.025	28.89 ± 2.34b	− 0.59	36.67 ± 2.15b	− 0.55
	0.050	13.33 ± 1.21c	− 0.81	20.00 ± 1.12c	− 0.75
	0.075	10.00 ± 0.26c	− 0.86	14.44 ± 0.34d	− 0.82
	0.100	0.00 ± 0.00d	− 1.00	0.00 ± 0.00e	− 1.00

Data are shown as the mean ± standard deviation. Different letters in the same column for each treatment indicate significant differences at the *P* < 0.05 level, while the same letter indicates no significant difference at the *P* < 0.05 level.

### Effects of the water extract from leaves of *Flaveria bidentis* on the seedling growth of three plants

Different concentrations of water extracts of *Flaveria bidentis* had an inhibitory effect on the seedling height and root length of the three plants (RI < 0), and as the extract concentration increased, the inhibitory effect gradually increased (Table 2). When the concentration was 0.025 g/mL, the seedling height and root length of Shanghai green, wheat and barnyard grass were significantly decreased compared with their respective controls (*P* < 0.05). When the concentrations were 0.050 g/mL and 0.075 g/mL, there was no significant difference between the two concentrations regarding the seedling height and root length of Shanghai green and wheat, but the seedling height and root length of barnyard grass were significantly different between the two concentrations. At a concentration of 0.100 g/mL, the inhibitory effect was the strongest, and the seedling height and root length of Shanghai green and barnyard grass both decreased to 0. At this time, the seedling height and root length of wheat decreased by 87.36% and 98.33%, respectively, compared with the control (Table 2).

**Table 2:** Effects of the water extract from leaves of *Flaveria bidentis* on the seedling height and root length of three plants.

Plant species	Concentration (g/mL)	Seedling height (mm)	RI of the seedling height	Root length (mm)	RI of the root length
Shanghai green	0	21.8 ± 1.41a		45.67 ± 2.89a	
	0.025	12.6 ± 1.12b	− 0.42	5.20 ± 0.13b	− 0.89
	0.050	2.87 ± 0.11c	− 0.87	1.53 ± 0.61bc	− 0.97
	0.075	1.03 ± 0.05c	− 0.95	0.07 ± 0.12c	− 0.99
	0.100	0.00 ± 0.00d	− 1.00	0.00 ± 0.00d	− 1.00
Wheat	0	32.67 ± 1.15a		31.67 ± 3.81a	
	0.025	15.67 ± 0.58b	− 0.52	4.67 ± 0.31b	− 0.85
	0.050	14.00 ± 1.36bc	− 0.57	2.27 ± 0.21b	− 0.93
	0.075	10.33 ± 1.52c	− 0.68	2.13 ± 0.10b	− 0.93
	0.100	4.13 ± 0.02d	− 0.87	0.53 ± 0.08c	− 0.98
Barnyard grass	0	74.33 ± 6.11a		61.67 ± 4.07a	
	0.025	43.33 ± 2.52b	− 0.42	21.67 ± 1.58b	− 0.65
	0.050	13.53 ± 1.81c	− 0.82	6.00 ± 0.58c	− 0.90
	0.075	5.27 ± 0.81d	− 0.93	1.67 ± 0.15d	− 0.97
	0.100	0.00 ± 0.00e	− 1.00	0.00 ± 0.00e	− 1.00

Data are shown as the mean ± standard deviation. Different letters in the same column for each treatment indicate significant differences at the *P* < 0.05 level, while the same letter indicates no significant difference at the *P* < 0.05 level.

### Effects of the leaf extract of *Flaveria bidentis* on the fresh and dry weights of three receptor plants

The four concentrations of leaf extracts had inhibitory effects on the fresh and dry weights of the three receptor plants (RI < 0), and the inhibitory effect increased as the concentration increased (Table 3). In terms of fresh weight, when the concentration was 0.025 g/mL, the fresh weight of Shanghai green and barnyard grass was significantly decreased compared with the control; although the fresh weight of wheat was decreased compared with the control, it did not reach a significant level. When the concentration was 0.050 g/mL-0.100 g/mL, the fresh weight of the three plants was significantly different from that of the control (Table 3). In terms of dry weight, when the concentration was 0.025 g/mL, the dry weight of barnyard grass was significantly different from that of the control, and the dry weight of Shanghai green and wheat was significantly decreased compared with that of the control, but this value did not reach a significant level. When the concentration was 0.050 g/mL, the dry weight of the three plants was significantly different from that of the control. When the concentration was 0.100 g/mL, the inhibition effect was the strongest, and the dry weight of Shanghai green, wheat and barnyard grass decreased by 94.59%, 55.71% and 92.86% compared with the control, respectively (Table 3).

**Table 3:** Effects of different concentrations of water extracts from the leaves of *Flaveria bidentis* on the fresh and dry weights of three plants.

Plant species	Concentration (g/mL)	Fresh weight (g)	RI of the fresh weight	Dry weight (g)	RI of the dry weight
Shanghai green	0	0.71 ± 0.07a		0.07 ± 0.02a	
	0.025	0.39 ± 0.04b	− 0.45	0.03 ± 0.01ab	− 0.57
	0.050	0.06 ± 0.01c	− 0.91	0.01 ± 0.01b	− 0.82
	0.075	0.02 ± 0.00c	− 0.97	0.01 ± 0.01b	− 0.85
	0.100	0.01 ± 0.00c	− 0.99	0.00 ± 0.00c	− 0.95
Wheat	0	1.50 ± 0.12a		0.42 ± 0.02a	
	0.025	1.02 ± 0.07ab	− 0.32	0.37 ± 0.02a	− 0.13
	0.050	1.01 ± 0.08b	− 0.33	0.29 ± 0.03b	− 0.31
	0.075	0.43 ± 0.08c	− 0.71	0.20 ± 0.01c	− 0.54
	0.100	0.31 ± 0.02c	− 0.79	0.19 ± 0.02c	− 0.56
Barnyard grass	0	0.67 ± 0.03a		0.14 ± 0.02a	
	0.025	0.23 ± 0.04b	− 0.66	0.09 ± 0.02b	− 0.36
	0.050	0.19 ± 0.02b	− 0.72	0.06 ± 0.01bc	− 0.57
	0.075	0.10 ± 0.01c	− 0.86	0.03 ± 0.00c	− 0.82
	0.100	0.01 ± 0.00d	− 0.98	0.01 ± 0.00d	− 0.93

Data are shown as the mean ± standard deviation. Different letters in the same column for each treatment indicate significant differences at the *P* < 0.05 level, while the same letter indicates no significant difference at the *P* < 0.05 level.

### Effect of the water extracts from the leaves of *Flaveria bidentis* on the chlorophyll content of three plants

Different concentrations of water extracts from the leaves of *Flaveria bidentis* had an inhibitory effect on the chlorophyll a (chl a) and chlorophyll b (chl b) contents of the three plants (RI < 0), and as the concentration of water extract increased, the inhibitory effect also increased (Table 4). For chl a, when the concentration was 0.025 g/mL, the chl a contents of Shanghai green and barnyard grass were significantly reduced compared with that of the control. The chl a content of wheat was decreased compared with that of the control, but this value did not reach a significant level. When the concentration was 0.075 g/mL, the chl a content of wheat was significantly different from that of the control. For chl b, when the concentrations were 0.025 g/mL and 0.050 g/mL, the chl b contents of Shanghai green, wheat and barnyard grass were significantly lower than that of the control, but no significant difference was reached. When the concentration was 0.075 g/mL, the chl b contents of Shanghai green, wheat and barnyard grass were significantly decreased compared with that of the control. When the concentration was 0.100 g/mL, the inhibitory effect was the most obvious, and the content of chl b also decreased to the minimum (Table 4).

**Table 4:** Effects of different concentrations of water extracts from the leaves of *Flaveria bidentis* on the chlorophyll a and chlorophyll b contents of three plants.

Plant species	Concentration (g/mL)	Chlorophyll a (mg/g)	RI of the chlorophyll a	Chlorophyll b (mg/g)	RI of the chlorophyll b
Shanghai green	0	0.96 ± 0.12a		0.38 ± 0.02a	
	0.025	0.89 ± 0.09b	− 0.07	0.36 ± 0.01a	− 0.05
	0.050	0.84 ± 0.11c	− 0.13	0.35 ± 0.03ab	− 0.07
	0.075	0.79 ± 0.04d	− 0.18	0.33 ± 0.01b	− 0.13
	0.100	0.78 ± 0.02d	− 0.19	0.32 ± 0.02b	− 0.15
Wheat	0	0.77 ± 0.10a		0.37 ± 0.02a	
	0.025	0.75 ± 0.07a	− 0.03	0.35 ± 0.03a	− 0.03
	0.050	0.72 ± 0.02a	− 0.06	0.34 ± 0.02ab	− 0.06
	0.075	0.67 ± 0.03b	− 0.13	0.33 ± 0.01bc	− 0.08
	0.100	0.64 ± 0.01b	− 0.17	0.32 ± 0.02c	− 0.12
Barnyard grass	0	0.86 ± 0.11a		0.37 ± 0.03a	
	0.025	0.81 ± 0.06b	− 0.06	0.36 ± 0.06a	− 0.03
	0.050	0.76 ± 0.07c	− 0.11	0.35 ± 0.05ab	− 0.06
	0.075	0.72 ± 0.05d	− 0.17	0.33 ± 0.02b	− 0.11
	0.100	0.70 ± 0.02d	− 0.19	0.32 ± 0.03b	− 0.14

Data are shown as the mean ± standard deviation. Different letters in the same column in each treatment indicate significant differences at the *P* < 0.05 level, while the same letter indicates no significant difference at the *P* < 0.05 level.

### Synthetical allelopathic effects of the water extract of *Flaveria bidentis* leaves on three plants

**Figure 1:**
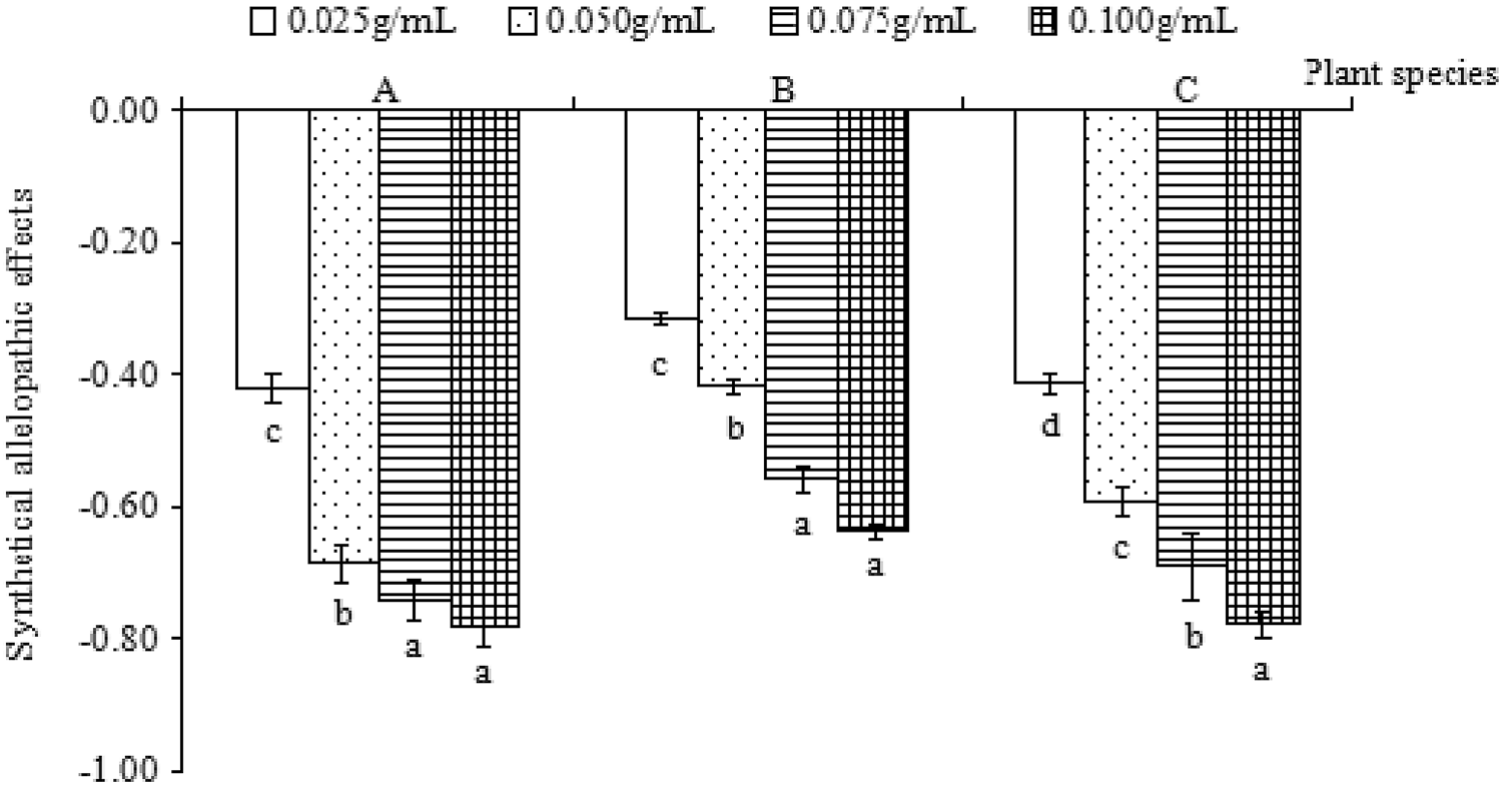
The synthetical allelopathic effects of *Flaveria bidentis* leaves on the three receptor plants. (**A**) Shanghai green, (**B**) Wheat, and (**C**) Barnyard grass.

The synthetical allelopathic effects (SE) of different concentrations of the water extract of *Flaveria bidentis* leaves on the three receptor plants were all inhibitory (SE < 0), and the inhibitory effect increased with an increase in the water extract concentration (Fig. 1). According to their absolute value, the allelopathic effects of the water extract of *Flaveria bidentis* leaves on the three receptor plants differed, and the order of the allelopathic effects on the three receptor plants was as follows: Shanghai green > barnyard grass > wheat. Wheat showed the strongest resistance to allelopathy (Fig. 1).

## Discussion and conclusion

The allelopathic effects of plants are mainly reflected in the germination of seeds, seedling height and root length of seedlings^23–26^. In the natural environment, allelochemicals are released into the environment and affect the surrounding plants. Water is the only solvent that can leach allelochemicals out of plants^27^. In this experiment, different concentrations of the water extracts from the leaves of *Flaveria bidentis* were used to treat the seeds of three different plants, Shanghai green, wheat and barnyard grass. The results showed that these water extracts from *Flaveria bidentis* had inhibitory effects on the germination potential, germination rate, seedling height and root length of the three plants, and the inhibitory effects increased as the concentration of the extract increased. This result was consistent with the study by Yan et al. on the allelopathic effect of *Flaveria bidentis* on four different crops^22^.

In addition, the results showed that the leaf extracts of *Flaveria bidentis* had inhibitory effects on the chlorophyll content, fresh weight and dry weight of seedlings of the three receptor plants. This result was consistent with the results of a preliminary study on the mechanism controlling the effect of *Flaveria bidentis* on the growth of several crops^28^. A previous study on the allelopathic effects of invasive *Flaveria bidentis* on cucumber came to similar conclusions as those of this experiment^20^. According to a comprehensive index, the allelopathic effects of *Flaveria bidentis* on three tested plants were found to differ. The allelopathic effects of *Flaveria bidentis* on the three plants from strong to weak were as follows: Shanghai green > barnyard grass > wheat. This result was similar to those from a study by Mustafa et al.^29^.

In summary, the water extract of *Flaveria Bidentis* leaves had prohibitive effects on the germination potential, germination rate, seedling height, root length, chlorophyll content, fresh weight and dry weight of three receptor plants, Shanghai green, wheat and barnyard grass, and the inhibitory effect increased as the extract concentration increased. Different receptor plants also had different allelopathic responses to the *Flaveria bidentis* leaf extract. The inhibitory effects of *Flaveria bidentis* extracts from strong to weak were as follows: Shanghai green > barnyard grass > wheat. Thus, wheat had the strongest resistance to the allelopathic effect of *Flaveria bidentis* among the three plants and could be planted in areas where *Flaveria bidentis* occurs. In addition, the water extracts from *Flaveria bidentis* obviously inhibited the seed germination and seedling growth of barnyard grass, suggesting that these extracts could be used as a biological control method for barnyard grass.

## Materials and methods

### Materials

The mature leaves of *Flaveria bidentis* plants were collected, with permission, from the *Flaveria bidentis* area on the west side of National Road 107 in the east section of Dongming Road, Xinxiang, China, in September 2018. The seeds of *Brassica rapa* L. (Shanghai Green) and *Echinochloa crus-galli* L. (barnyard grass) were purchased from the Institute of Agricultural Science in Xinxiang. Wheat seeds (Bainong207) came from the wheat breeding center of Henan Institute of Science and Technology.

### Preparation of the water extract from *Flaveria bidentis* leaves

The leaves were cleaned with distilled water, cut into pieces with scissors, and air dried naturally. Then, 50 g of the leaves was weighed and placed into a 1000 mL conical flask; then, 500 mL of distilled water was added, and the leaves were soaked at 4 °C for 48 h. The leachate was filtered with double-layer gauze to obtain the mother liquor (a water extract of *Flaveria bidentis* leaves) at 0.1 g/mL and stored at 4 °C for later use. Distilled water was added to dilute the extract to 0.025, 0.050 and 0.075 g/mL.

### Seed germination test

Healthy, plump and uniform seeds were selected and disinfected with 10% sodium hypochlorite solution for 3 min and then washed with distilled water 3 times. The treated seeds were put into similar-sized petri dishes, and a specific amount of seeds was placed into the dishes (wheat, 20 seeds per dish; Shanghai green, 50 seeds per dish; and barnyard grass, 30 seeds per dish). 10 mL of different concentrations of extracts were added to each petri dish, and 10 mL of distilled water was added to the control. Each treatment was repeated three times. Culturing was conducted at room temperature (approximately 25 °C), and the light and dark period was L/D = 12 h/12 h.

During the experiment, the number of germinated seeds in each dish was counted every 24 h (taking the radicle breaking through the seed coat to a length of 1 mm as the germination standard), and the extract was added over time. In the germination test, the last count was made when no new seeds germinated for 3 consecutive days. After 10 days, 10 seedlings were randomly selected from each dish to measure seedling height, the longest root length, fresh weight and dry weight. The germination potential and germination rate were calculated according to number of germinated seeds counted every day.

$$ {\text{Germination}}\;{\text{potential}}\;\left( \% \right) = \left( {{\text{Number}}\;{\text{of}}\;{\text{ germinated}}\;{\text{seeds}}\;{\text{in}}\;{\text{the}}\;{\text{first}}\;{5}\;{\text{days}}/{\text{total}}\;{\text{ number}}\;{\text{of}}\;{\text{test}}\;{\text{seeds}}} \right) \times {1}00\% , $$

$$ {\text{Germination}}\;{\text{ rate}}\; \, \left( \% \right) = \left( {{\text{Final}}\;{\text{number}}\;{\text{of}}\;{\text{germinated}}\;{\text{seeds}}/{\text{total}}\;{\text{number}}\;{\text{of}}\;{\text{test}}\;{\text{seeds}}} \right) \times {1}00\% . $$

### Determination of the chlorophyll content

Plant leaves were removed, cleaned with distilled water, weighed to 0.2 g, and put into a test tube. Then, 10 mL of 95% ethanol was added, and the tubes were placed in a dark room for 48 h, with 3 rounds of shaking during the extraction. The extract was poured into a colorimetric cup, and the absorbance was measured at 665 nm and 649 nm, with 95% ethanol as a blank. The contents of chlorophyll a (chl a) and chlorophyll b (chl b) were calculated.

$$ \begin{aligned} {\text{Chl a }} & = { 13}.{\text{95 A665}}\;{\text{ nm}} - {6}.{\text{88 A649}}\;{\text{ nm}} \\ {\text{Chl b }} & = { 24}.{\text{96 A649 }}\;{\text{nm}} - {7}.{\text{32 A665}}\;{\text{ nm}} \\ \end{aligned} $$

The formula for calculating the pigment content in the leaves is as follows:

$$ {\text{Chlorophyll }}\;{\text{content}}\; \, \left( {{\text{mg}}/{\text{g}}} \right) = \left( {{\text{Concentration }}\;{\text{of}}\;{\text{ chlorophyll }} \times {\text{ total }}\;{\text{volume}}\;{\text{ dilution}}\;{\text{ of}}\;{\text{ the}}\;{\text{ extract}}} \right)/{\text{fresh}}\;{\text{ weight }}\;{\text{of }}\;{\text{the }}\;{\text{sample}}. $$

### Analysis of allelopathic effects

The allelopathic response index (RI) is a measurement index proposed by Williamson and Richardson^30^ to measure allelopathic intensity. RI = 1 − C/T(T ≥ C) or RI = T/C − 1 (T < C), where C is the corresponding index value of the control and T is the corresponding index value of the treatment. RI > 0 indicated that there was a promoting effect, RI < 0 indicated that there was an inhibiting effect, and the absolute value of RI indicated the strength of the allelopathy. The synthetical allelopathic effects (SE) were evaluated using the average RI value of 7 indices, germination potential, germination rate, seedling height, longest root length, fresh weight, dry weight and chlorophyll content, of the same receptor plant under the same treatment^31^^.^. All methods were carried out in accordance with relevant guidelines.

### Data analysis

The experimental data were processed and analyzed using SPSS 18.0 (Statistical Product and Service Solutions, International Business Machines Corporation, USA) and Excel 2010 for statistical analysis and graphing. The measured results were averaged, and their standard deviation was determined. Differences between groups with *P* < 0.05 were regarded as statistically significant.
